# Social media use and weight bias internalization: association moderated by age and weight perception

**DOI:** 10.1186/s40337-024-01043-7

**Published:** 2024-06-18

**Authors:** Michelle Moufawad, Asef Hoque, Meredith Kells, Kendrin R. Sonneville, Samantha L. Hahn

**Affiliations:** 1 Central Michigan University College of Medicine, 600 East Preston St, Mount Pleasant, MI 48859 USA; 2 University of Rochester School of Nursing, Rochester, NY 14642 USA; 3 University of Michigan School of Public Health, Ann Arbor, MI 48109 USA

**Keywords:** Social media, Weight bias, Weight perception, Body image, College women

## Abstract

**Background:**

The current study examined whether weight perception or age moderated associations between time spent on image-based social media and weight bias internalization (WBI).

**Methods:**

Data come from the baseline visit of the Tracking Our Lives Study, a randomized control trial of college women (*n* = 200). Participants completed questionnaires assessing time spent on social media (continuous, overall and individual platforms Instagram, Facebook, and Snapchat), WBI (continuous), weight perception (perceive their weight as “overweight” vs. do not perceive their weight as “overweight”), age (continuous, 18–49 years), and confounders (race/ethnicity, parent education, sexual orientation, and BMI). Adjusted zero-inflated Poisson regressions were performed to determine if weight perception and age moderated associations between time spent on image-based social media and WBI.

**Results:**

As expected, we found a positive association between overall time spent on image-based social media and WBI (β = 0.826, *p* < 0.001). In moderation analyses, the strength of the association was weakened among women who perceived their weight as “overweight” (β=-0.018, *p* = 0.006). Associations also weakened with age (β=-0.001, *p* < 0.001). The association between time spent on Instagram and WBI was also weakened with age (β=-0.014, *p* = 0.018), which was the only significant moderation found for individual social media platforms.

**Conclusions:**

Our results suggest that image-based social media use is more strongly associated with increases in WBI among younger women.

**Supplementary Information:**

The online version contains supplementary material available at 10.1186/s40337-024-01043-7.

## Abstract (plain language summary)

There is research reporting that college women who spend an increased amount of time on image-based social media apply more negative-based weight stereotypes to oneself, leading to weight-based self-degradation. This is known as weight bias internalization. However, there may be things that make social media more or less harmful for college women. The goal of this study was to see if a woman’s age or their perception of their weight changed the relationship between the amount of time spent on image-based social media and weight bias internalization. Data come from 200 college women who completed questionnaires to determine time spent on social media, their level of weight bias internalization, how they perceived their weight, age, and other demographics. Results showed that women who perceive their weight as “overweight” had a weaker association between time spent on all image-based social media and weight bias internalization, as did older women. Our results suggest that younger women may be more susceptible to influences on social media that increase their weight bias internalization. These results can be used to identify those who may benefit from interventions aimed at reducing weight bias internalization and prevent later health consequences as a result of having more weight bias internalization.

**Supplementary Information:**

The online version contains supplementary material available at 10.1186/s40337-024-01043-7.

## Background

Image-based social media use, like Instagram, Facebook, and Snapchat, is common among American young-adults, and many individuals spend hours each day using image-based social media [1, 2]. Nearly 71% of young adults aged 18–29 use Instagram, 70% use Facebook, and 65% use Snapchat [1]. The impact of image-based social media use on weight bias internalization (WBI), the application of negative-based weight stereotypes to oneself, leading to self-degradation [3], has been well documented [4, 5]. WBI is an often overlooked public health issue that impacts daily activities [6], social interactions [7], and even health care choices [8]. Use of image-based social media may lead to increases in WBI because the images viewed on social media promote an “ideal” body image that is unrepresentative of the majority of women [9, 10]. One study found that viewing images of ultra-fit thin-ideal body types leads to increased WBI and higher rates of body dissatisfaction amongst college-aged women compared to women who viewed images of “normal” weight, ultra-fit models [11]. Women who compare themselves to edited, idealized images of women on social media perceive a greater discrepancy between their appearance and that of others [12]. While emerging literature suggests that content viewed on social media is a more salient predictor of body image disturbance than overall time spent on social media [13], there are a range of structural, interpersonal, and intrapersonal experiences within image-based social media platforms [4, 5] that may contribute to time-dependent increases in WBI [14]. Specifically, with increasing time spent on platforms, algorithms may be more likely to show content that is stigmatizing or reinforces stigmatizing norms [15] or people may be more likely to experience cyberbullying based on their weight [16].

While the relationship between social media use and WBI has been well documented, the extent to which individual characteristics, such as weight perception or age, may influence the association is understudied. Weight perception is defined by how individuals view their body weight, regardless of whether it matches their actual weight. Research has found that women who perceive their weight as “overweight” had significantly lower levels of moderate physical activity and lower levels of exercise enjoyment [6], changes in social behavior due to embarrassment [7], and higher rates of disordered eating [17]. Those who perceive themselves as “overweight” also have higher levels of WBI, irrespective of actual weight status [18]. It is possible that the association between image-based social media use and WBI would differ by weight perception because of how social media content was internalized, which may differ based on the extent to which someone identifies as being “overweight”.

Age could also impact the associations between image-based social media use and WBI. As mentioned above, young adults aged 18–29 frequently use image-based social media. Particularly for individuals just transitioning to college, this is likely the first time they are living independently and experiencing drastic changes in their living, social, and eating environments during this transitional period. This may result in those transitioning to college spending more time on image-based social media to connect with peers, seek out information on eating and food, etc. or being more susceptible to the harmful messages incurred on social media [19].

Therefore, the current study serves to understand whether weight perception and age moderate the associations between image-based social media use and WBI among college women. We hypothesized that those who perceive their weight as “overweight” would have a stronger association between time spent on image-based social media and WBI compared to those who did not perceive their weight as “overweight”. Secondly, we hypothesized that age would negatively moderate the association between time spent on image-based social media and WBI, in that older college women would experience a weaker association between image-based social media and WBI.

## Methods

### Study population & design

Participants were enrolled in previously described Tracking Our Lives Study, a randomized controlled trial that examined dietary self-monitoring and eating disorder risk in college women [20]. The study population included 200 undergraduate women who had daily access to a smartphone, were fluent in English, and were at least 18 years of age. Exclusion criteria included a self-reported history of any medical condition that impacts the type or amount of food eaten, reported dietary self-monitoring in the last year, a current or previous eating disorder diagnosis or an Eating Disorder Examination-Questionnaire Short mean score ≥ 2, indicating high eating disorder risk. Recruitment took place through an email to a random sample of women through the Office of the Registrar. After completing screening, a total of 411 students were eligible to participate. Based on parent study a priori power analyses, 201 students were invited to participate in the study. One participant was excluded due to a deviation in study protocol, resulting in a final sample size of 200. More descriptive information about the study design can be found elsewhere [20].

Data from the present study includes baseline data from the randomized control trial. Study visits occurred in person at the Behavioral Nutritional Lab at the University of Michigan. Participants gave written informed consent and completed a survey. Trained staff then measured participant height (cm) and weight (kg). All data was collected using REDCap. The study was determined to be exempt from oversight by the University of Michigan Institutional Review Board.

### Measures

#### Sociodemographic characteristics

Participants completed self-reported sociodemographic measures including race and ethnicity, parent education, and sexual orientation. BMI was calculated using participant height and weight. Age was calculated based on birthdays recorded at the initial visit.

#### Image-based social media use

Social media use was measured by asking, “Thinking back over the past week, how much time did you usually spend on __ on a typical day? Include total time spent on all devices.” The question was asked for Facebook, Instagram, Snapchat, and Twitter, but since this study focuses on image-based media, Twitter was excluded from analysis. Answers included “0 minutes,” “Less than half an hour,” “Between a half an hour to 1 hour,” “More than 1 hour, but less than 2 hours,” “More than 2 hours, but less than 3 hours,” “More than 3 hours, but less than 4 hours,” and “More than 4 hours” for each question. Each image-based social media was treated as a continuous outcome using the midpoint of response options and then treated as the number of hours per week, as has been done prior in similar studies of social media use [21, 22]. A combined variable was also created for total image-based social media use per week. Time spent on individual platforms and combined time was assessed to determine if there was an overall association, and to examine if there were differences based on each platform. We elected to examine individual platforms as well as overall image-based social media use given the prior research indicating differences between individual platforms on related constructs (i.e., body satisfaction and appearance comparison) [23].

#### Weight perception

Participants were asked, “How would you describe your weight?” Response options included, “very underweight”, “slightly underweight”, “about the right weight”, “slightly overweight”, and “very overweight”. Responses were dichotomized into two categories: perceive their weight as “overweight” and do not perceive their weight as “overweight”. Those that answered, “very underweight”, “slightly underweight”, and “about the right weight” were considered to not perceive their weight as “overweight.” Participants that answered, “slightly overweight” and “very overweight” were considered to perceive their weight as “overweight”, in line with other weight perception literature which compares those who perceive their weight as “overweight” versus those who do not perceive their weight as “overweight” [8, 24, 25].

#### Weight bias internalization (WBI)

WBI was evaluated using a five question validated scale [26]: “I am concerned that other people’s opinion of me will be based on my weight,” “I am worried that most people will judge me on the basis of my weight,” “I am concerned that I will not be treated fairly by others because of my weight,” “I am afraid that other people will reject me because of my weight,” and “I am concerned that others will not respect me because of my weight.” Participants responded using a scale from 0 to 4 for each question, with 0 indicating “never” and 4 indicating “all the time.” Participants’ five scores were summed for a final score out of 20 with higher scores indicating higher levels of WBI. Cronbach’s Alpha analysis was performed for these questions to determine consistency of individual scores (α = 0.93).

### Statistical analysis

Pearson’s chi-square test and Mann-Whiteny U analyses were performed to test associations between sociodemographic factors and weight perception. Mann-Whitney U tests were performed to examine whether there were differences in amount of time spent on image-based social media use and WBI between those that do and do not perceive themselves as “overweight.” Zero-Inflated regressions were performed to examine the relationship between amount of time spent on image-based social media use (independent variable) and WBI (dependent variable) for the moderation analysis, adjusting for race/ethnicity [27], parent education [28], sexual orientation [29], and BMI [30]. Confounders were selected a priori based on use of a directed acyclic graph and existing literature showing differences in social media use and/or WBI by these variables [27–30]. Zero-inflated negative binomial regression was used due to the non-normal zero-skewed distribution for the WBI variable. Analyses were performed for overall time spent on image-based social media and for each of the individual platforms (Facebook, Instagram, and Snapchat). Overall time spent on image-based social media use was log-transformed because of non-normal distribution. A *p*-value of ≤0.05 was considered statistically significant for all analyses. Analyses were performed using SPSS 28.0 (Armonk, NY). Spearman’s rank correlations were run among all continuous variables to assess correlations between study variables included as Supplemental Table 1.

## Results

### Sample characteristics

Overall, the study population was predominantly White (50.5%), their parents were highly educated, and 81% of participants identified as heterosexual; more specific demographic information can be found in Table 1. Those that perceived themselves as “overweight” had a higher median BMI (26.0 Interquartile range [IQR]: 24.6–29.3) compared to those that did not perceive themselves as “overweight” (median = 20.8, IQR:19.6–22.3, *p* < 0.001). There was a significant difference between those who perceive their weight as “overweight” and do not perceive their weight as “overweight” by parent education (*p* = 0.006) and sexual orientation (*p* = 0.019). There is no significant difference between the two groups in terms of median age (*p* = 0.0.66) and race/ethnicity (*p* = 0.724).

**Table 1:** Baseline characteristics of study population overall and by weight perception.*

	Overall	Perceive Their Weight as “Overweight” (%)	Do Not Perceive Their Weight as “Overweight”	*p*-value
*N* (%)				
**Overall**	200	64 (32.0)	136 (8.0)	
**Race/Ethnicity**				
Asian or Pacific Islander	58 (29.0)	17 (29.3)	41 (70.7)	0.724
Black or African American	13 (6.50)	-	-	
Hispanic/Latina	12 (6.00)	-	-	
Mixed/Other	14 (7.00)	-	-	
White	101 (50.5)	33 (32.7)	68 (67.3)	
**Parent Education**				
High school education or less or GED equivalent	19 (9.50)	-	-	**0.006**
Some College, Associates or other 2-year college	31 (15.5)	16 (51.6)	15 (48.4)	
Bachelor’s degree	50 (25.0)	19 (38.0)	31 (62.0)	
Advanced degree (master’s or higher)	100 (50.0)	20 (20.0)	80 (80.0)	
**Sexual Orientation**				
Bisexual	27 (13.5)	9 (33.3)	18 (67.7)	**0.019**
Gay/Lesbian, Questioning, or Another Sexual Orientation	11 (5.5))	-	-	
Straight	162 (81.0)	47 (29.0)	115 (70.0)	
**Body Mass Index (kg/m**^**2**^)				
< 18.5	16 (8.00)	-	-	**< 0.001**
18.5–24.9	135 (67.5)	21 (15.6)	114 (84.4)	
25.0-29.9	33 (16.5)	28 (84.8)	5 (15.20)	
≥ 30.0	16 (8.00)	-	-	

Median (Interquartile Range)				
**Body Mass Index (kg/m**^**2**^)	21.8 (20.0–25.0)	26.0 (24.6–29.3)	20.8 (19.6–22.3)	**< 0.001**
**Age (yrs)**	20.1 (19.1–20.9)	20.3 (19.5–21.0)	19.9 (19.0-20.9)	0.066

*All values presented as n (%) or median (interquartile range). Two-sided *p*-values are reported and results were considered significant if *p* < 0.05 (bolded). Cells with less than 10 people were not presented to protect confidentiality of participants and are presented as “ - ” within cells.

### Social media and weight bias internalization univariate and bivariate analyses

In the overall sample, participants spent a median average of 10.5 hours per week on social media across all image-based social media was (IQR: 7.0–21.0); there was no significant difference in median amount of time spent on image-based social media per week for those that perceive their weight as “overweight” (median = 14.0, IQR: 10.5–23.6) and those that do not perceive their weight as “overweight” (median = 10.5, IQR:7.0–21.0, *p* = 0.136). (Table 2). There was no significant difference in time spent on Facebook, Instagram, and Snapchat for those who perceive their weight as “overweight” and those that do not perceive their weight as “overweight”. The median WBI was significantly higher for those who perceive their weight as “overweight” (median = 5.0, IQR: 1.0-8.8) compared to those that do not perceive their weight as “overweight” (median = 1.0, IQR: 0.0-2.8, *p* < 0.001).

**Table 2:** Social media use and weight bias internalization by weight perception.*

	Overall	Perceive Their Weight as “Overweight”	Do Not Perceive Their Weight as “Overweight”	
	Median (IQR)			*p*-value
**Social media use (hours in past week)**				
Overall Image Based Social Media	10.50 (7.0–21.0)	14.0 (10.5–23.6)	10.5 (7.0–21.0)	0.136
Facebook	0.0 (0.0-3.50)	3.50 (0.0-6.13)	0.0 (0.0-3.50)	0.067
Instagram	3.50 (0.0–7.0)	3.50 (0.0-12.25)	3.50 (0.0–7.00)	0.921
Snapchat	3.50 (0.0–7.00)	3.50 (0.0–7.00)	3.5 (0.0–7.00)	0.612
**Weight Bias Internalization (score 0–20)**	1.00 (0.0–5.00)	5.00 (1.00-8.75)	1.00 (0.0-2.75)	**<0.001**

*All values presented as median (interquartile range). Median time spent on social media and WBI was compared between groups with and without perceiving “overweight” using Mann-Whitney U tests. Two-sided *p*-values are reported and results were considered significant if *p* < 0.05 (bolded).

### Adjusted associations between social media and weight bias internalization; weight perception moderation

In regression models that included both main effects (overall image-based social media time and weight perception), and an interaction term between weight perception and image-based social media time, we found that there was a positive association between total time on image-based social media and WBI (β = 0.826, SE = 0.206, *p* < 0.001, Table 3). There was also a significant positive association between weight perception and WBI (β = 0.143, SE = 0.039, *p* < 0.001). We also found evidence of moderation, such that the association between image-based social media and WBI was weaker among those that perceive their weight as “overweight” compared to those who did not perceive their weight as “overweight” (β=-0.018, SE = 0.006, *p* = 0.006).

In analyses for specific image-based social media platforms, we found similar positive associations for all main effects other than for Snapchat (*p* = 0.254). However, we did not find any evidence of moderation by weight perception in the association between time spent on individual image-based social media platforms.

**Table 3:** Associations between social media use and weight bias internalization, moderation by weight perception.*

	Social Media Main Effects			Weight Perception Main Effects			Social Media x Weight Perception		
	β	SE	*p*-value	β	SE	*p*-value	β	SE	*p*-value
Overall social media	0.826	0.206	< 0.001	0.143	0.039	< 0.001	-0.018	0.006	0.006
Facebook	0.031	0.031	0.028	0.711	0.134	< 0.001	-0.009	0.016	0.576
Instagram	0.021	0.009	0.030	0.790	0.149	< 0.001	-0.021	0.012	0.073
Snapchat	0.011	0.010	0.254	0.724	0.144	< 0.001	-0.017	0.014	0.205

^*^ A Zero-Inflated Poisson Regression was used to analyze the relationship between social media use and WBI. Covariates include race/ethnicity, parent education, sexual orientation, BMI, and age. Weight perception was included as a moderator. The overall social media use variable was log transformed for comparison to the variable in the age analysis. *p*-values less than 0.05 indicate significant associations between social media use and WBI and an interaction between social media and weight perception (bolded).

### Adjusted associations between social media and weight bias internalization; age moderation

In the model examining overall time spent on image-based social media, we found that WBI increased as time spent on social media increased (β = 1.444, SE = 0.351, *p* < 0.001, Table 4) and age increased (β = 0.169, SE = 0.041, *p* < 0.001). The association between total time spent on image-based social media and WBI was moderated by age such that as age increased, the association between the time spent on overall image-based social media and WBI decreased (β=-0.001, SE = 0.00, *p* < 0.001).

When examining individual image-based social media platforms, we found that there was evidence of moderation by age for amount of time spent on Instagram such that the association between amount of time spent on Instagram and WBI decreased with age (β=-0.014, SE = 0.006, *p* = 0.018). No other evidence of moderation by age was found for Facebook or Snapchat.

**Table 4:** Associations between social media use and weight bias internalization, moderation by age.*

	Social Media Main Effects				Age Main Effects			Social media x Age		
	β	SE	*p*-value		β	SE	*p*-value	β	SE	*p*-value
Overall social media	1.444	0.351	< 0.001		0.169	0.041	< 0.001	-0.001	0.000	< 0.001
Facebook	0.228	0.194	0.239		0.135	0.047	0.004	-0.010	0.009	0.290
Instagram	0.294	0.125	0.018		0.190	0.051	< 0.001	-0.014	0.006	0.018
Snapchat	-0.014	0.168	0.932		0.113	0.064	0.078	0.001	0.008	0.918

*A Zero-Inflated Poisson Regression was used to analyze the relationship between social media use and WBI. The model was adjusted for race/ethnicity, parent education, sexual orientation, BMI, and weight perception. Age was included as a moderator. Overall social media was log-transformed because of a non-normal distribution. *p*-values less than 0.05 indicate significant associations between social media use and WBI and interactions between social media and age (bolded).

## Discussion

The present study aimed to examine the associations between image-based social media use and WBI among college women and explore whether associations were moderated by weight perception and age. Our results indicated that counter to our hypothesis, a weaker association between overall image-based social media time and WBI was seen among those who perceive their weight as “overweight”. In congruence with our hypothesis, the relationship between overall image-based social media use and WBI was weakened as age increased. When examining specific image-based social media platforms, the only significant moderation was that increased age also weakened the association between amount of time spent on Instagram and WBI. Understanding the impact of weight perception and age on the associations between time spent on image-based social media and WBI will allow for a more robust understanding of the complex associations between image-based social media and women’s health, including identifying populations for future interventions.

In both moderation analyses, time spent on overall image-based social media was positively associated with WBI, as seen in previous literature [12, 31]. In a study conducted by Hogue & Mills (2019), young women that actively engaged with image-based social media of attractive peers were found to have a more negative body image. Body image is defined as the mental description one has of their physical self; though it is not the same as weight perception, they are both describing how individuals perceive their bodies. The association found between increased time on image-based social media and altered body perception by Hogue & Mills (2019) led us to hypothesize that the interaction between image-based social media and perceiving one’s weight as “overweight” would increase WBI. Unexpectedly, we found that perceiving one’s weight as “overweight” weakened the association between overall image-based social media time and WBI. This may be because women who perceive their weight as “overweight” experience greater amounts of weight stigma outside of image-based social media, which reduces the impact of image-based social media on their WBI. For example, weight-related comments from mothers and weight-based teasing both significantly predict adolescents’ WBI [32]. Further, individuals with larger bodies who experienced stigma for their weight as children but became “normal weight” as adults were at greater risk of psychological distress as adults [33]. Because women who perceive their weight as “overweight” experience greater levels of weight stigma from external sources, which impacts their WBI, it could be that consuming image-based social media does not have as potent effects. Additionally, the type of content consumed by participants was not specified, so it is unknown if it was different than that observed by those who do not perceive their weight as “overweight”. It is also possible that the fact that our sample was restricted to those that had not dietary self-monitored in the last year and were low-risk for an eating disorder may have impacted results. Individuals who perceive their weight as “overweight” often engage in weight loss attempts [34], and many individuals use self-monitoring to do so [35]. Therefore, our sample may be unique in that even if they perceive their weight as “overweight,” they may be inherently less likely to have been trying to lose weight than the general population of individuals who perceive their weight as “overweight”. Findings should therefore be replicated in a broader audience that is not restricted based on self-monitoring or eating disorder risk. Further mechanistic research is necessary to confirm what factors contribute to WBI in women who perceive their weight as “overweight” and if results are replicated in other samples.

Individually, time spent on both Facebook and Instagram had a significant positive association with WBI in regression analyses, but Snapchat did not. The null Snapchat finding is surprising because of the multitude of filters available to enhance facial features, which have led to “Snapchat Dysmorphia” [36]. While filters and photo-editing similar to Snapchat have been associated with higher rates of desired cosmetic surgery [9, 37], this has mainly been demonstrated for facial changes. It may be that the facial focus of Snapchat, rather than whole body, does not impact WBI. It may also be due to the time available for images in Snapchat (i.e. temporary, disappear quickly), compared to other platforms. Similarly, it could be due in part to whose content is being viewed on Snapchat (e.g., close friends) compared to other platforms (e.g., celebrities, influencers, etc.). In contrast with the facially-focused content of Snapchat, there is greater variability in content posted on Instagram and Facebook, including posted or photoshopped images of the whole body. A study found that girls spending more time on the internet had higher rates of internalization of thin-ideals, body surveillance, and drive for thinness [38]. More specifically, Facebook users scored higher on these three measures compared to girls who did not use the platform. While the study did not specifically measure WBI, internalization of thin-ideals, body surveillance and drive for thinness are closely related. They all develop from the belief that a thin body type is ideal and desirable. Users of social media are overexposed to individuals who exemplify the thin ideal, which leads to regular comparison of the user’s body and the thin ideal, increasing the rate of body dissatisfaction and drive for thinness [39]. Therefore, significant positive associations between time spent on Instagram and Facebook and WBI were expected.

Age significantly negatively moderated the association between time spent on image-based social media and WBI, as hypothesized for both overall time and time spent on Instagram. Though generally considered the same age group, it is important to determine the differences amongst different years of college. Studies indicate that the mean age of onset for different eating disorders correlates with different ages within college, with the mean age of onset for anorexia being 19 years old [40]. For many college freshman, this is the first time living independently, with their social setting and eating habits changing dramatically. The “freshman 15” phenomena is a popular unsubstantiated belief that individuals gain 15 pounds during their first year of college [41]. The phenomena concerns many female freshman, though studies indicate that weight gain (if any) in the first year of college are not that dramatic [42]. However, the fear of excessive weight gain, pressure to make new friends and other changes in social and environmental pressures could make early-college females more susceptible to increases in WBI from thin-ideal exposure on image-based social media. This may indicate that interventions focusing on reducing health impacts of image-based social media use among college students should target those transitioning to target to have maximal public health impact.

A limitation of this study is the sample size. It is possible that the sample size may not have been large enough to see statistical significance in the moderation analyses. Relatedly, there were differences in correlation and regression results (e.g., time spent on Instagram was not significant in correlational analyses but was significant in regression analyses); therefore, findings should be interpretated with caution and future research is warranted to explain this phenomenon among a larger sample. Secondly, not all image-based social media platforms were assessed. For example, our analysis did not include TikTok as it rose in popularity shortly after data collection ended and warrants further research given the video content and advertising for weight loss supplements, exercise, and “What I Eat in a Day” videos, the platform could greatly contribute to WBI [43]. We also may have missed less common image-based social media platforms by not including an “Other” option to the questionnaire. Moreover, because we used a Likert scale to assess the amount of time spent on social media sites, future studies should use objective measures to examine the nuances of amount of time spent on social media. It could also be beneficial to further examine nuances of types of social media engagement, as there is evidence that specific content of social media consumed and ways of interacting with apps may impact associations between social media use and health outcomes, which we were unable to examine in the present study [5]. Data was also collected from a predominantly White and fairly affluent institution, and most participants were heterosexual; therefore, results may not encompass the experience of all college women. Further, associations need to be examined in gender diverse populations and in non-college and more age diverse populations. Despite differences within this age group, our study population was restricted to college women and results cannot be generalized to different age groups. While limiting our research to college students allowed us to identify a population that could be feasibly targeted for future intervention (i.e., college freshmen), it does hamper our ability to draw conclusions across diverse age ranges such as adolescents, who are highly susceptible to body ideals and weight bias as they undergo puberty; therefore, future research is needed to examine whether our results hold true in a larger age range, particularly adolescents. Results may also not be generalizable to those who have high eating disorder risk, as our sample was restricted to those with low baseline eating disorder risk and no history of self-monitoring. Future research should therefore consider looking at associations among a sample that also includes high risk individuals. Lastly, the data on social media use was self-reported, potentially leading to bias.

## Conclusions

This is the first study, to the authors’ knowledge, to demonstrate that weight perception and age may moderate associations between image-based social media use and WBI. College women who perceive their weight as “overweight” have a weaker association between time spent on overall image-based social and WBI. Older aged college women also experienced a weakened association. Further studies are necessary to confirm this relationship and explore potential mechanisms in other populations. It is possibly the result of factors outside of image-based social media that have a stronger influence on women who perceive their weight as “overweight”. Better elucidating moderating variables on the association between time spent on image-based social media and WBI can lead to more targeted interventions to reduce potential harmful effects of social media use among college women.

### Electronic Supplementary Material

Below is the link to the electronic supplementary material.

Supplementary Material 1

## Data Availability

No datasets were generated or analysed during the current study.

## Abbreviations

WBI: Weight Bias Internalization
