# Supplementary material for: Social media use and weight bias internalization: association moderated by age and weight perception
Source: J Eat Disord. 2024 Jun 18;12:84. doi: 10.1186/s40337-024-01043-7 (PMC11186141; doi:10.1186/s40337-024-01043-7)
Supplement: Supplementary file 1 — Supplementary Material 1 [file 40337_2024_1043_MOESM1_ESM.docx]

| **Supplemental Table 1**. Spearman’s Rank Correlation Test Results | | | | | | | | |
| --- | --- | --- | --- | --- | --- | --- | --- | --- |
| Variable | Weight Bias Internalization | Total Image-Based Social Media | Facebook | Instagram | Snapchat | Age | BMI |  |
| Weight Bias Internalization | -- | 0.158* | 0.227** | 0.127 | 0.090 | 0.099 | 0.310** |  |
| Total Image-Based Social Media |  | -- | 0.464** | 0.809** | 0.700** | -0.083 | 0.064 |  |
| Facebook |  |  | -- | 0.163* | 0.071 | 0.173* | 0.058 |  |
| Instagram |  |  |  | -- | 0.456** | -0.083 | -0.010 |  |
| Snapchat |  |  |  |  | -- | -0.265** | 0.006 |  |
| Age |  |  |  |  |  | -- | 0.169* |  |
| BMI |  |  |  |  |  |  | -- |  |
| **p-value <0.01, *p-value<0.05. | | | | | | | | |
